# P-976. The Effectiveness of Augmented Reality Handouts for Learning Microbiology in Medical Students

**DOI:** 10.1093/ofid/ofae631.1166

**Published:** 2025-01-29

**Authors:** Nuntra Suwantarat, Vorawan Vanichcharoenchai, Pongsagon Vichitvejpaisal, Kasana Raksamani

**Affiliations:** Chulabhorn International College of Medicine, Thammsat University Hospital, Thammasat University, Maung, Pathum Thani, Thailand; Faculty of Nursing, Mahidol University, Bangkok Noi, Krung Thep, Thailand; Deputy Director of the International Program on Digital Design & Digital Technology Bachelor's and Masters’ Degree Program, King Mongkut's University of Technology Thonburi, Bangkok, Krung Thep, Thailand; Faculty of Medicine, Siriraj Hospital, Mahidol univeristy, Bangkok Noi, Krung Thep, Thailand

## Abstract

**Background:**

Augmented reality (AR) is a novel technology-enabled active learning for medical education. The goal of this study was to determine effectiveness of self-directed learning AR handouts as a learning material for teaching medical microbiology in the 3rd year medical students, Doctor of Medicine (English Program), Thammasat University.

**Figure:**
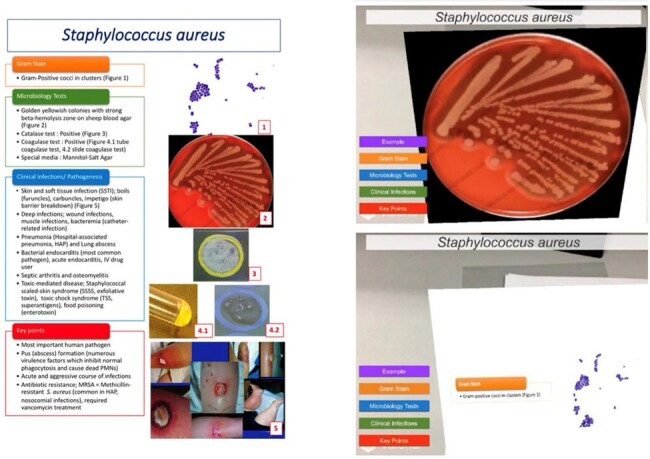

Example of the traditional handouts and the AR handouts in this study

**Methods:**

A randomized controlled trial study was conducted by comparing academic performances (MCQs scores), the motivation level (Motivation Scores Learning Questionnaire, MSLQ) and satisfaction survey scores in the students after studied AR handouts (intervention group) and traditional handouts (control group). The content of AR handout was similar to the traditional handout (topic: common Gram-positive bacteria).

**Figure:**
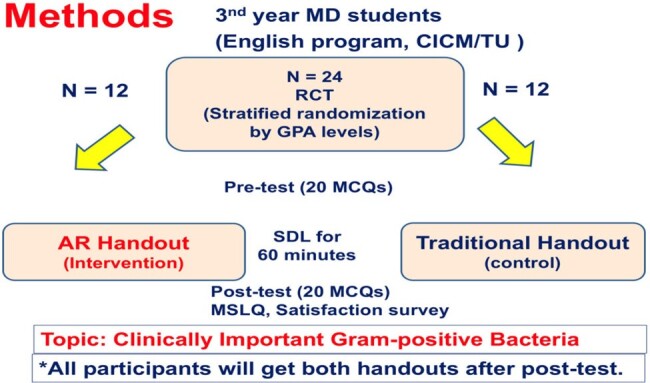

Study Procedures and Methods

**Results:**

Twenty-four students were enrolled in this study (n = 02 each group). The both groups had no differences on age, gender, total GPA, pre-test scores (means of 5.08). The both groups also had similar increases in the post-test scores means of 11.25 (intervention group) and 10.58 (control group). MSLQ (topic of intrinsic goal orientation) was significantly higher in the intervention group than the control group (5.73 vs 4.81, p = 0.002). Satisfaction survey scores (topic of "Handouts are stimulating" and "Handouts are exciting") were significantly higher in the intervention group than the control group (4.58 vs 3.50, p = 0.025 and 4.42 vs 3.17, p = 0.019).

**Conclusion:**

AR handouts for learning medical microbiology and infectious diseases was effective to improve the knowledge in medical students. Based on cognitive information theory, the AR handouts help learning process by increased students' motivation, and satisfaction.

**Disclosures:**

**All Authors**: No reported disclosures

